# The effects of rising atmospheric carbon dioxide on shoot-root nitrogen and water signaling

**DOI:** 10.3389/fpls.2013.00304

**Published:** 2013-08-09

**Authors:** Hsien Ming Easlon, Arnold J. Bloom

**Affiliations:** Department of Plant Sciences, University of California at DavisDavis, CA, USA

**Keywords:** carbon dioxide, nitrogen, nitrate assimilation, water, drought, salinity, chilling

## Abstract

Terrestrial higher plants are composed of roots and shoots, distinct organs that conduct complementary functions in dissimilar environments. For example, roots are responsible for acquiring water and nutrients such as inorganic nitrogen from the soil, yet shoots consume the majority of these resources. The success of such a relationship depends on excellent root–shoot communications. Increased net photosynthesis and decreased shoot nitrogen and water use at elevated CO_2_ fundamentally alter these source–sink relations. Lower than predicted productivity gains at elevated CO_2_ under nitrogen or water stress may indicate shoot–root signaling lacks plasticity to respond to rising atmospheric CO_2_ concentrations. The following presents recent research results on shoot–root nitrogen and water signaling, emphasizing the influence that rising atmospheric carbon dioxide levels are having on these source–sink interactions.

## INTRODUCTION

Land plants occupy highly dissimilar aboveground and belowground environments and face the basic allocation dilemma of where to invest resources (Bloom et al., 1985). Too little investment in roots leads to nutrient- or water-limited growth, whereas too much investment compromises shoot growth, reproduction, and photosynthesis. Excellent communications between roots and shoots are paramount for meeting the immediate demands of distal organs to optimize resource supply from them, while avoiding superfluous distribution of resources.

For example, the dependence of photosynthesis on nitrogenous compounds and the inevitability of water loss during CO_2_ uptake (Field and Mooney, 1986) makes communicating N and water availability from roots to shoots essential to maintain shoot productivity (Boyer, 1982; Bloom, 1997). Conversely, shoot to root communication of leaf N status is necessary to optimize carbohydrate allocation in roots among growth, N uptake, and inorganic N assimilation. Coordination of N transport from root to shoot and of carbohydrate transport from shoot to root is fundamental for maintaining a C/N ratio throughout the plant that is optimal for plant growth and development (Martin et al., 2002; Zheng, 2009).

Climate change, in particular rising CO_2_, is likely to alter root–shoot communications. Atmospheric CO_2_ concentrations have remained relatively low, between 180 and 300 μmol mol^-^^1^ over the last 400,000 years (Petit et al., 1999) and between 140 and 320 μmol mol^-^^1^ over the last 23 million years (Pearson and Palmer, 2000). Flowering plants have evolved specific adaptations to this low CO_2_ environment including increased stomatal density (Beerling and Chaloner, 1993), increased leaf vein density (Boyce and Zwieniecki, 2012), and C_4_ photosynthesis (Ehleringer et al., 1991). This concentration has increased from 280 to 400 μmol mol^-^^1^ since 1800 from the burning of fossil fuels (Whorf and Keeling, 1998) and is projected to reach between 500 and 900 μmol mol^-^^1^ by the end of the century (Joos et al., 1999). This CO_2_ enrichment will increase photosynthesis in C_3_ plants and will decrease shoot N and water requirements for photosynthesis. This frequently results in increased biomass and productivity in the short-term that is not sustained in the long-term (Dukes et al., 2005; Korner, 2006; Kimball et al., 2007). Only after long-term growth at elevated CO_2_ do limitations from N deficiencies, carbohydrate transport, and altered shoot/root allocation patterns become apparent. Unknown is whether the mechanisms of long distance communication between roots and shoots that evolved under low CO_2_ will have the plasticity to optimize coordination of root and shoot growth under long-term exposure to elevated CO_2_.

The goal of this review is to describe shoot–root signaling for N and water and to examine the observed and predicted responses of these signaling mechanisms to rising atmospheric CO_2_ concentrations. First, we discuss shoot–root N signaling, changing C and N demand, and the breakdown of N signaling at elevated CO_2_. Then, we explore the common and distinctive features of drought, salinity, chilling, and high vapor pressure deficit and the opposing effects of elevated CO_2_ on chemical and hydraulic water stress signaling. Finally, we consider the effects of non-optimal shoot–root coordination on plant growth at elevated CO_2_.

## NITROGEN: COMMUNICATING ROOT AVAILABILITY AND SHOOT DEMAND

For most plants, growth and productivity is highly dependent upon N obtained from root absorption of soil inorganic and organic N. In most temperate soils, the primary form of N available to plants is nitrate (${NO}_{3}^{-}$ ; Epstein and Bloom, 2005). Therefore, this review focuses on this form.

Many studies have shown that elevated CO_2_ stimulates photosynthesis, plant growth, and demand for mineral nutrients. High variability in plant growth and photosynthetic responses to elevated CO_2_ may result from vast experimental differences in soil ${NO}_{3}^{-}$ concentration. In natural systems, soil ${NO}_{3}^{-}$ is typically around 1 mM (Andrews, 1986b), but in fertilized agricultural soils, ${NO}_{3}^{-}$ can be much higher, ranging from 10 to 70 mM (Reisenauer, 1966). The negative charge of ${NO}_{3}^{-}$ prevents it from binding to most soil particles, and this contributes to substantial spatial and temporal heterogeneity in soil ${NO}_{3}^{-}$ availability (Jackson and Caldwell, 1993). Plants have responded to soil ${NO}_{3}^{-}$ variability with adaptations to increase ${NO}_{3}^{-}$ uptake rapidly when it is available. In response to high soil ${NO}_{3}^{-}$ , individual roots increase ${NO}_{3}^{-}$ uptake (Forde, 2002a) and alter root hydraulic properties to increase mass flow (Gorska et al., 2008). These adaptations allow a few roots in a high ${NO}_{3}^{-}$ region of the soil to provide all the N that the shoot requires (Laine et al., 1995).

## ROOT TO SHOOT N SIGNALING

Root to shoot communication of soil N availability may be as simple as ${NO}_{3}^{-}$ delivery from roots to shoots in xylem sap (Takei et al., 2002). When soil ${NO}_{3}^{-}$ is low, root C/N ratios are high and roots have sufficient carbohydrate to assimilate most of the ${NO}_{3}^{-}$ that they absorb (Andrews et al., 1992) and thus deliver little ${NO}_{3}^{-}$ to shoots. As soil ${NO}_{3}^{-}$ increases, a greater proportion of absorbed ${NO}_{3}^{-}$ remains unassimilated in the root and is transported to the shoot (Andrews, 1986a; Agrell et al., 1994). Xylem sap ${NO}_{3}^{-}$ directly links soil N availability to the shoot and thereby serves as an ideal signal for such a temporally and spatially variable nutrient. High shoot ${NO}_{3}^{-}$ stimulates shoot growth and low shoot ${NO}_{3}^{-}$ inhibits shoot growth even when total shoot N is high (Walch-Liu et al., 2000; Rahayu et al., 2005). Species that predominantly transport N from root to shoot as amino acids instead of ${NO}_{3}^{-}$ may not use xylem sap ${NO}_{3}^{-}$ for root to shoot N signaling (Sprent and Thomas, 1984). Indeed, leaf growth is not always proportional to leaf ${NO}_{3}^{-}$ concentration (Rahayu et al., 2005), indicating the importance of other signals such as phytohormones for root to shoot communication of root N supply.

One class of phytohormones involved in root to shoot signaling is cytokinins. Stimulation of leaf growth by N supply is associated with increased concentrations of active forms of cytokinins (Rahayu et al., 2005). Root cytokinin production and xylem sap delivery of cytokinins to shoots increase with ${NO}_{3}^{-}$ fertilization (Takei et al., 2001; Forde, 2002b). Cytokinins stimulate leaf growth, increase shoot sink strength (Werner et al., 2008), and delay leaf senescence (Gan and Amasino, 1995), while they inhibit root elongation. Xylem sap transport of cytokinins increases expression of N responsive genes in leaves (Sakakibara et al., 1999; Takei et al., 2001; Kiba et al., 2011; Ruffel et al., 2011). All of these responses to cytokinins suggest that these phytohormones serve as root to shoot signals for root N availability

## ELEVATED CO_2_ EFFECTS ON ROOT TO SHOOT N SIGNALS

CO_2_ enrichment influences root to shoot N signaling through its effects on xylem sap flow rate, ${NO}_{3}^{-}$ assimilation, and root allocation.

Root to shoot signals of N availability depend upon xylem sap flow for rapid signal delivery, and elevated CO_2_ affects xylem flow rates. Elevated CO_2_ decreases transpiration rates between 5 and 20% as stomata close in response to higher intercellular CO_2_ concentration (Leakey et al., 2009). Stomatal closure slows water uptake and thereby xylem sap flow rate. Decreased transpiration may impede mass flow of ${NO}_{3}^{-}$ in the soil solution to roots (McDonald et al., 2002), but this decrease may not slow delivery of N to shoots (Schulze and Bloom, 1984) because N concentration in the xylem sap increases as xylem sap flow decreases, maintaining N delivery rates (Shaner and Boyer, 1975; Schulze and Bloom, 1984). Increasing xylem loading of N in roots does not require substantial energy in that xylem solute N concentrations are relatively low. Xylem concentrations of cytokinins are in the nanomolar range (Foo et al., 2007), and so are even less likely to be affected by xylem sap flow rates.

Elevated CO_2_ may disrupt root to shoot N signaling through shifting the location of ${NO}_{3}^{-}$ assimilation. Greater rates of photosynthesis at elevated CO_2_ increase carbohydrate flux to roots (Grimmer and Komor, 1999). In the root, higher carbohydrates increase ${NO}_{3}^{-}$ assimilation (Matt et al., 2001), growth, and local demand for N (Kircher and Schopfer, 2012). Consequently, the root transports less ${NO}_{3}^{-}$ to the shoot, and xylem sap ${NO}_{3}^{-}$ becomes less effective as a signal of root N availability.

Plant allocation of carbohydrate to roots varies greatly with CO_2_ enrichment (Rogers et al., 1996). For species in which carbohydrate flux to roots is insensitive to CO_2_, the relationship among root ${NO}_{3}^{-}$ assimilation, root N utilization, and xylem sap ${NO}_{3}^{-}$ transport could indicate the potential for improving root to shoot N signaling at elevated CO_2_. For species in which CO_2_ enrichment increases carbohydrate flux, elevated CO_2_ may disrupt cytokinin signaling. A low baseline level of root cytokinin production at low root available ${NO}_{3}^{-}$ (Samuelson and Larsson, 1993) may result in greater root xylem cytokinin loading when root allocation is high under long-term growth at elevated CO_2_ (Yong et al., 2000). High rates of cytokinin delivery to shoots could induce shoot growth in excess of what can be supported by root N supply. This could partially explain the decline in leaf N after prolonged exposure to elevated CO_2_ (Oren et al., 2001). Additional study of xylem sap and leaf cytokinins at elevated CO_2_ are necessary to determine if this disruption in cytokinin signaling is responsible for declining leaf N content.

## SHOOT TO ROOT N SIGNALING

When soil ${NO}_{3}^{-}$ is high, a few roots – 3.5% of the root system in spring wheat (Robinson et al., 1991) and 12% in lettuce (Burns, 1991) – can supply leaves with all of their N. When leaf N becomes limiting, plants may enhance root uptake by increasing (1) root growth, (2) root transporters to absorb soil N, and (3) root exudation to stimulate soil microbe activity that accelerates mineralization (Hawkes et al., 2005). All of these N acquisition strategies expend carbohydrate exported from shoots, and coordination of these processes is essential for optimal plant growth. Signals that stimulate root growth when leaf N is low or that repress root growth when leaf N is high balance root N acquisition and shoot demand.

A significant portion of N transported to shoots is recycled to roots via phloem transport of amino acids (Forde and Clarkson, 1999). It has been hypothesized that this transport of amino acids from shoots to roots in phloem could allow for feedback inhibition of root growth and ${NO}_{3}^{-}$ assimilation (Marschner, 1986; Imsande and Touraine, 1994; Marschner et al., 1996). Although exogenously supplied amino acids can inhibit root growth and ${NO}_{3}^{-}$ uptake (Orsel et al., 2002; Forde and Walch-Liu, 2009), composition and transport of amino acids in phloem often do not correlate with shoot N status or root ${NO}_{3}^{-}$ uptake (Forde, 2002a). In split root experiments, amino acids were preferentially transported to portions of root systems supplied with ${NO}_{3}^{-}$ rather than those deprived of exogenous N, and the roots receiving more amino acids had higher growth rates (Tillard et al., 1998). This supports that amino acids delivered via the phloem stimulate root growth rather than inhibit it (Marschner et al., 1996).

Auxins are primarily synthesized in shoots and inhibit shoot branching (Normanly et al., 1995; Ljung et al., 2001). They are transported to roots through polar transport in the phloem (Baker, 2000) and promote proliferation of lateral roots. Phloem and root auxin concentrations decrease when plants are grown at high ${NO}_{3}^{-}$ (Caba et al., 2000; Tian et al., 2008) and increase in roots when N is limiting (Walch-Liu et al., 2006). Therefore, auxins are prime candidates for signals that communicate shoot ${NO}_{3}^{-}$ levels to roots (Forde, 2002b). Roots rely on photosynthesizing organs for carbohydrates, and thus, auxin-induced increases in root growth depend upon root carbohydrate supply (Reed et al., 1998; Bhalerao et al., 2002; Zhang et al., 2007).

The amount of carbohydrate transported in phloem sap from shoots to roots may also signal shoot N status, and this carbohydrate signaling mechanism appears to be independent of phloem transport of auxin (Bingham et al., 1998). At high leaf N, shoot growth acts as a sink for shoot produced carbohydrates and relatively little carbohydrate is transported to roots. If leaf N is low, shoot growth is limited and more carbohydrate is transported to roots (Brouwer, 1967; Brouwer and DeWit, 1969; Bloom et al., 1993; Kallarackal et al., 2012). High root carbohydrates increases root elongation and lateral root initiation (Bingham et al., 1998; Kircher and Schopfer, 2012), increases root area for N acquisition, and upregulates ${NO}_{3}^{-}$ uptake and assimilation (Lejay et al., 1999; Ono et al., 2000; Matt et al., 2001).

## ELEVATED CO_2_ EFFECTS ON SHOOT TO ROOT N SIGNALING

Leaf N concentrations decline under prolonged growth at elevated CO_2_ (Oren et al., 2001). Photosynthetic acclimation can account for some of this decrease (Long et al., 2004), but fertilization with NH_4_NO_3_ eliminates it (Crous et al., 2010; Liu et al., 2011), showing that increased N supply can compensate for the effects of elevated CO_2_ through enhanced root N uptake and plant N assimilation. This suggests that elevated CO_2_ interrupts shoot to root N signaling.

Amino acids in the phloem, potential signals of shoot N status, do not show a consistent response to elevated CO_2_ (Docherty et al., 1997; Sicher, 2008). By contrast, leaf and root auxins increase under elevated CO_2_ and stimulate root growth (Teng et al., 2006; Wang et al., 2009; Niu et al., 2011). Other processes, however, such as carbohydrate transport or shoot ${NO}_{3}^{-}$ assimilation, may limit the ability of increased root auxins to stimulate root N uptake.

Carbohydrate transport through the phloem is driven by a carbohydrate concentration gradient (van Bel, 2003). Higher rates of net photosynthesis under elevated CO_2_ increase carbohydrate delivery to roots and can increase root respiration and root ${NO}_{3}^{-}$ assimilation (Bassirirad et al., 1996; Fonseca et al., 1997; Kruse et al., 2002). High carbohydrate delivery to roots of C_3_ plants under long-term growth at elevated CO_2_ can also increase root growth (Berntson and Bazzaz, 1996; Kimball et al., 2002) and root carbohydrate exudation (Berntson et al., 1997). Carbohydrate flow from shoots to roots, however, does not increase proportionally to photosynthesis at elevated CO_2_. For example, elevated CO_2_ increases photosynthesis in C_3_ species, but carbohydrate export from the leaves may not increase proportional to this carbon fixation (Grodzinski et al., 1998). This probably derives from leaf carbohydrate production under elevated CO_2_ exceeding phloem export capacity (Korner et al., 1995; Komor, 2000).

In most tropical and subtropical plants and in temperate plants at high soil ${NO}_{3}^{-}$ , most ${NO}_{3}^{-}$ assimilation occurs in shoots because ${NO}_{3}^{-}$ photoassimilation in shoots is more energy efficient than respiratory-driven ${NO}_{3}^{-}$ and ${NO}_{2}^{-}$ reduction in roots (Andrews, 1986b). Elevated CO_2_ inhibits shoot ${NO}_{3}^{-}$ assimilation in C_3_ plants (Rachmilevitch et al., 2004; Bloom et al., 2010), necessitating a greater reliance on root ${NO}_{3}^{-}$ assimilation to maintain plant capacity for ${NO}_{3}^{-}$ assimilation. In tobacco, 3 weeks of CO_2_ enrichment enhances root ${NO}_{3}^{-}$ assimilation and may compensate for decreasing shoot ${NO}_{3}^{-}$ assimilation when there is sufficient root carbohydrate (Kruse et al., 2002). A shift from shoot ${NO}_{3}^{-}$ assimilation to root ${NO}_{3}^{-}$ assimilation requires translocation of more carbohydrate to the roots to provide sufficient energy and carbon skeletons for these processes (Zheng, 2009). ${NH}_{4}^{+}$ fertilization decreases the limitations of phloem carbohydrate transport on plant N status because ${NH}_{4}^{+}$ assimilation requires less carbohydrate.

## WATER STRESS SIGNALING

Photosynthesis in land plants results in the inevitable water loss during CO_2_ uptake because both diffusion of CO_2_ into leaves and water vapor out of leaves occur through stomata. Soil drought, salinity, and chilling can result in an inability of water transport from roots to match shoot water loss. To maintain leaf photosynthesis, shoot turgor, and shoot growth, plants under water stress rely on local root responses that increase water uptake as well as shoot responses that reduce water use.

During drought or salt stress, xylem tension acts as an integrative hydraulic signal of soil water potential that rapidly communicates soil water stress to leaves (Malone, 1993). Likewise, low root hydraulic conductance during root chilling results in rapidly increasing xylem tension and declining leaf turgor (Bloom et al., 2004). Turgor loss causes stomatal closure through either passive or active regulation (Tardieu and Davies, 1993) and inhibits leaf growth as leaf cell turgor declines below the threshold for cell wall expansion (Hsiao and Acevedo, 1974). Smaller leaf area and stomatal closure resulting from decreased leaf turgor protect leaves from desiccation. During slowly developing soil drought, soil moisture content has substantial heterogeneity, but hydraulic signals are integrative; that is, xylem tension in leaves is affected by xylem tension in all connected roots. Roots in drier regions experience greater decreases in water potential before hydraulic signals are transmitted to leaves. Non-hydraulic signals can be generated in these roots with lower water potential, allowing shoots to preemptively reduce shoot water use before leaf water deficit develops (Dodd et al., 2008). During root chilling, chilling tolerant species close stomata before declines in leaf water potential occur, indicating that non-hydraulic chemical signals are also important in response to this type of water stress (Bloom et al., 2004).

Abscisic acid (ABA) increases with drought and salinity, induces stomatal closure, and inhibits transpirational water loss (Davies and Zhang, 1991; Bahrun et al., 2002; Jia et al., 2002). Low root water potential increases both root ABA production (Simonneau et al., 1998) and xylem sap transport of ABA from root to shoot (Zhang and Davies, 1989). ABA production also increases during chilling stress in the long-term (Melkonian et al., 2004), but the rapidity of stomatal closure during root chilling indicates that other, more rapidly produced root to shoot signals are involved in root chilling.

Abscisic acid-induced stomatal closure is not solely dependent on root ABA production. Shoot vascular tissue ABA production (Endo et al., 2008) and ABA uptake by leaf symplast also affect guard cell ABA concentration. Xylem sap pH increases with soil drought, salinity, and root chilling, slows leaf symplastic ABA uptake, and increases guard cell ABA concentration, thereby promoting stomatal closure (Vernieri et al., 2001; Wilkinson and Davies, 2002; Felle et al., 2005; Wilkinson et al., 2007).

Evidence is mounting for non-hydraulic signals other than ABA and pH in xylem sap that also affect stomatal regulation during water stress (Munns, 1992; Chen et al., 2002; Holbrook et al., 2002). For example, salts carried in the transpiration stream can also act as long distance root to shoot signals. During salinity stress Na^+^ and Cl^-^ are transported in xylem sap and concentrated at sites of evaporation in leaves. High leaf apoplastic Na^+^ and Cl^-^decrease water potential, prompting osmotic adjustment and, in some halophytes, stomatal closure (Very et al., 1998).

Shoot to root signaling is also important for responses to chilling and high vapor pressure deficit stresses that do not directly affect root water potential. During both of these stresses, transpiration exceeds the capacity for root water transport. High root ABA increases root hydraulic conductance and water flow during chilling or at high vapor pressure deficit to ameliorate shoot water deficit (Markhart, 1984; Kudoyarova et al., 2011). This increase in root ABA requires water stress signaling from shoots; for example, if leaf water potential is maintained during chilling, there is no increase in root ABA (Vernieri et al., 2001). Shoot to root communication of shoot water deficits may be communicated hydraulically or through phloem transport of ABA or other signals.

## ELEVATED CO_2_ EFFECTS ON WATER STRESS SIGNALING

The primary effect of elevated CO_2_ on water stress signaling derives from stomatal closure in response to high intercellular CO_2_ and the resulting lower transpiration rates (Leakey et al., 2009). Lower transpiration rates under elevated CO_2_ may decrease both accumulation of ABA at sites of evaporation near guard cells (Zhang and Outlaw, 2001) and foliar ABA concentration in general (Teng et al., 2006). Moreover, stomatal closure in response to root ABA application and osmotic stress are greater at elevated CO_2_ (Leymarie et al., 1999) and may result from higher intercellular CO_2_. At ambient CO_2_, when stomata begin to close during water stress, low intercellular CO_2_ can partially reverse stomatal closure. At elevated CO_2_, intercellular CO_2_ remains high even after stomatal closure, and this can prevent reversal of stomatal closure.

Hydraulic signaling is also affected by lower transpiration rates at elevated CO_2_. Slower transpiration reduces leaf xylem tension and improves leaf water potential during drought (Xiao et al., 2005). This may mitigate midday declines in leaf water potential during early stages of drought that are necessary for shoot perception of water stress. Slower transpiration at elevated CO_2_ delays hydraulic signaling of declining root water potential, but does not delay non-hydraulic signaling. Non-hydraulic signals like ABA are still delivered to shoots at elevated CO_2_, decreasing shoot water use and further delaying hydraulic signaling of declining root water potential. Slower transpiration also minimizes development of leaf water deficit during chilling at elevated CO_2_ (Boese et al., 1997), which may inhibit root ABA production (Vernieri et al., 2001) that is important for root acclimation to chilling.

## CONCLUSION

Leaf N concentration declines under prolonged growth at elevated CO_2_ (Oren et al., 2001) unless plants are heavily fertilized with NH_4_NO_3_ (Crous et al., 2010; Liu et al., 2011). This suggests that mechanisms for long distance root–shoot communication of root N availability and shoot N status, which evolved under low CO_2_, may lack plasticity to maintain root–shoot coordination under elevated CO_2_. Leaf and root auxin concentrations increase in response to low leaf N under elevated CO_2_ which should increase root growth, root ${NO}_{3}^{-}$ uptake, and root ${NO}_{3}^{-}$ assimilation (Teng et al., 2006; Wang et al., 2009; Niu et al., 2011). However, root organic N supply to shoots may be limited by phloem carbohydrate transport from shoots to roots (Grodzinski et al., 1998); although these effects may not affect growth until stored leaf N is depleted. The accumulation of non-structural carbohydrates in leaves at elevated CO_2_ that is often observed (Long et al., 2004) may result from an inability to transport carbohydrate out of leaves or to obtain enough N from roots for shoot growth. Photosynthetic acclimation, whereby carbon fixation per unit leaf area declines under prolonged exposure to elevated CO_2_, decreases leaf N requirements and increases leaf phloem export capacity. This may mitigate phloem carbohydrate export limitations and thus improve shoot–root N signaling.

The improvement in leaf water potential and water use efficiency resulting from higher intercellular CO_2_ concentration are predicted to benefit plant growth under elevated CO_2_, but productivity gains at elevated CO_2_ under water limitation are often lower than predicted (Nowak et al., 2004; Newingham et al., 2013). Slower transpiration impedes development of leaf water deficits important for shoot water stress perception as soil water potential declines. Plants generate ABA and other non-hydraulic signals of low root water potential, and these can decrease stomatal conductance and shoot growth before declines in leaf water potential occur. While stomatal closure from these non-hydraulic water stress signals has less negative impact on photosynthesis at elevated CO_2_ as compared to ambient CO_2_, these signals can still unnecessarily limit shoot growth (Leymarie et al., 1999). Greater stomatal sensitivity to osmotic and drought stress results in high water use efficiency and less negative leaf water potential, but more conservative shoot growth and lower potential productivity (Warren et al., 2011).

Shoot–root N and water signaling involve both resource and phytohormone transport from source organs to distant sink organs to achieve a functional equilibrium between roots and shoots. Rising atmospheric CO_2_ concentrations will increase net photosynthesis, decrease water use, and may alter source–sink interactions beyond the capability of signaling mechanisms that evolved at the lower atmospheric CO_2_ concentrations, which have prevailed throughout recent history (**Table 1**). Critical assessment of limitations in shoot–root signaling at elevated CO_2_ and careful genetic manipulations of N and water signaling could enhance crop response to rising atmospheric CO_2_ and avoid declines in plant N.

**Table 1:** Root–shoot N and water signal responses to elevated CO_2_.

Signal	Role	Response to elevated CO_2_
${NO}_{3}^{-}$	Root to shoot signal of root ${NO}_{3}^{-}$ availability	Root ${NO}_{3}^{-}$ assimilation, local root demand for N increase, and xylem transport of ${NO}_{3}^{-}$ decreases
Cytokinin	Root to shoot signal of root ${NO}_{3}^{-}$ availability	Cytokinin production and xylem transport increases even at low root available ${NO}_{3}^{-}$
Auxin	Shoot to root signal of leaf N availability	Auxin production and transport to roots increases in response to low leaf N
Carbohydrate	Shoot to root signal of leaf N availability	Increased carbohydrate delivery to roots, but delivery does not increase proportionally with leaf carbohydrate production
Xylem tension	Bidirectional signal of root or shoot water stress	Stomatal closure reduces leaf xylem tension delaying shoot perception of water stress
ABA	Bidirectional signal of root or shoot water stress	Transpirational accumulation of leaf and guard cell ABA decreases and stomatal sensitivity to ABA increases

## Conflict of Interest Statement

The authors declare that the research was conducted in the absence of any commercial or financial relationships that could be construed as a potential conflict of interest.
